# Showing the limitations of available phenotypic assays to detect *Burkholderia pseudomallei* from clinical specimens in Nigeria

**DOI:** 10.1099/acmi.0.000604.v5

**Published:** 2023-10-18

**Authors:** Oluwatosin Qawiyy Orababa, Solayide A. Adesida, Rebecca F. Peters, Zainab AbdulGanniyu, Olawale Olakojo, Adefunke Abioye

**Affiliations:** ^1^​ Department of Microbiology, Faculty of Science, University of Lagos, Akoka, Nigeria; ^2^​ Department of Medical Microbiology and Parasitology, Lagos University Teaching Hospital, Idi-Araba, Lagos, Nigeria; ^3^​ Lagos State Biobank, Mainland Hospital, Yaba, Lagos, Nigeria; ^†^​ Present address: School of Life Sciences, Gibbet Hill campus, University of Warwick, Coventry, UK

**Keywords:** *Burkholderia pseudomallei*, Ashdown’s agar, Gram-negative, phenotypic, antimicrobial susceptibility, Nigeria

## Abstract

The genus *Burkholderia* comprises Gram-negative bacteria that are metabolically complex and versatile, often thriving in hostile settings. *Burkholderia pseudomallei *, the causative agent of melioidosis, is a prominent member of the genus and a clinical pathogen in tropical and sub-tropical regions. This pathogen is well known for its multidrug resistance and possible bioweapon potential. There is currently no report of the pathogen from clinical specimens in Nigeria, which might be due to misdiagnosis with phenotypic assays. This study aims to explore the accuracy of the use of phenotypic assays to diagnose *B. pseudomallei* in Nigeria. Two hundred and seventeen clinical samples and 28 Gram-negative clinical isolates were collected and analysed using Ashdown’s selective agar and monoclonal antibody-based latex agglutination. Species-level identification was achieved using the analytical profile index (API) 20NE system. The susceptibility of the isolates to nine different antimicrobial agents was determined using the disc diffusion method. A total of seventy-four culture-positive isolates were obtained using Ashdown’s selective agar. Twenty-two of these isolates were believed to be *B. pseudomallei* through the monoclonal antibody-based latex agglutination test and the API 20NE system subsequently identified 14 isolates as *Burkholderia *. The predominant *Burkholderia* species was *B. cepacia* with an isolation rate of 30.8 % (8/26). No isolate was distinctively identified as *B. pseudomallei* but five isolates were strongly suspected to be *B. pseudomallei* with similarity indices ranging from 81.9–91.3 %. Other bacterial species with definitive identity include *Aeromonas* sp., *Sphingomonas* sp. and *Pseudomonas aeruginosa *. The antibiotic susceptibility results revealed an overall resistance to amoxicillin–clavullanic acid of 71.4 %, to cefepime of 33.3 %, to trimethoprim–sulfamethoxazole of 38.1 %, to piperacillin–tazobactam of 33.3 %, to imipenem of 66.7 %, to doxycycline of 57.1% and to ceftazidime of 66.7 %. The highest intermediate resistance was observed for cefepime and piperacillin–tazobactam with a value of 66.7 % each, while there was no intermediate resistance for gentamicin, colistin and imipenem. Our findings, therefore, show that phenotypic assays alone are not sufficient in the diagnosis of melioidosis. Additionally, they provide robust support for present and future decisions to expand diagnostic capability for melioidosis beyond phenotypic assays in low-resource settings.

## Data Summary

The authors confirm that all supporting data and protocols have been provided within the article or as supplementary data files (Data S1, available in the online version of this article). Data S1 contains demographic data for the samples analysed in this study, and the raw biochemical identification results (both presumptive and API 20NE).

## Introduction

The bacterial genus *Burkholderia* houses Gram-negative organisms that are known to infect both plants and animals [1]. Members of this genus are metabolically complex and versatile, often thriving in hostile settings [2–4]. Popular members of the group include *B. cepacia *, *B. mallei *, *B. thailandensis* and *B. pseudomallei. B. pseudomallei*, the causative agent of melioidosis, is an environmental pathogen known to inhabit both soil and water in sub-tropical and tropical regions, especially in Southeast Asia and Northern Australia [5–8]. The potential of *B. pseudomallei* for use as a bioterrorism agent earns it its classification as a category B agent (second highest priority) by the Centers for Disease Control and Prevention (CDC) [9, 10].

*B. pseudomallei* has been reported in both humans and animals from different parts of Africa [11–13]. However, to the best of our knowledge, there has been no report of this pathogen from clinical samples in Nigeria despite the country being predicted to have a comparable incidence of melioidosis to that of India, Indonesia and Bangladesh [14]. The only report of melioidosis from a Nigerian was in the UK from a Nigerian woman who had visited Nigeria shortly before she was diagnosed with the infection [15]. The true source of the infection was not clear and might have suggested that the pathogen was picked up during her visit to Nigeria, especially because the UK’s weather conditions do not favour the proliferation of the pathogen. Despite the scarcity of data on the prevalence of the pathogen in clinical samples in Nigeria, the pathogen has been reported in non-clinical samples in the country [16, 17].

It can be argued that the lack of data on the clinical occurrence of the pathogens in the country might be due to misdiagnosis. Although standard laboratory media support the growth of *B. pseudomallei *, specialized agar such as Ashdown’s agar, *B. cepacia* selective agar and *B. pseudomallei* selective agar are best used for its potential diagnosis [18, 19]. Unfortunately, these media are not readily available in many diagnostic laboratories in low-resource settings such as Nigeria. Additionally, phenotypic biochemical assays – the most readily available in low-resource settings such as Nigeria – often misidentify *B. pseudomallei* [20]. Collectively, these factors may have contributed to the lack of evidence for the clinical roles of the organism in our hospitals, and the magnitude of its involvement in clinical infections remains to be elucidated. In view of this, we hypothesize that the bacterium may be present and causing infections in our hospitals and we aim to explore the efficiency of the use of phenotypic assays in its identification from clinical specimens.

## Methods

### Study design and specimen collection

This is a cross-sectional study. Two hundred and seventeen clinical specimens from patients and 28 isolates representing a range of non-lactose-fermenting Gram-negative bacilli were examined in this study. All clinical specimens were received and collected by the laboratory department of the participating hospitals and included respiratory secretions, urine, throat swab, sputum and faeces. The sputa had been analysed for tuberculosis bacilli and tested negative using GeneXpert. Both the clinical specimens and isolates were collected over a period of 3 months from Lagos State University Teaching Hospital (LASUTH), Lagos University Teaching Hospital (LUTH) and the Nigerian Institute for Medical Research (NIMR). The age and gender of the patients whose samples or isolates were collected were retrieved from the laboratory records. The study was approved by the institutional Health Research Ethical Committee (reference no. NHREC/19/08/2019B).

### Specimen processing and preliminary bacterial identification

The specimens were inoculated onto MacConkey and blood agar for the isolation of non-lactose-fermenting Gram-negative bacilli. Plates were incubated at 37 °C for 24 h [21]. The primary identification was made with basic microbiological methods using colony morphology, Gram staining, catalase indole tests, motility tests, oxidase reaction and catalase tests. All isolates were then subcultured on Ashdown’s agar and the plates were incubated at 37 °C for 24–48 h. The colony appearance on Ashdown’s media was noted. The distinctive purple colour and dry and wrinkled texture of *B. pseudomallei* colonies can be used to provide a preliminary diagnosis [22]. Suspected *Burkholderia* isolates were subcultured on tryptic soy agar (Himedia) for purity.

### Latex agglutination test

A latex agglutination test kit (Mahidol University, Thailand) was used as recommended by the manufacturers. Briefly, approximately 10 µl of the latex reagent containing latex particles coated with monoclonal antibody against *B. pseudomallei* antigen was pipetted onto a clean glass slide. A sterile inoculating loop was then used to pick the bacterial cell and emulsified in the latex reagent. The slide was subjected to gentle rocking for 2 min, after which the reaction was recorded as either positive (agglutination) or negative (no agglutination). *B. thailandensis* was used as a negative control. Unfortunately, there was no positive control as standard *B. pseudomallei* strains were not available and could not be purchased for safety reasons.

### Identification using API 20NE biochemical test panel

A cell suspension of a 0.5 McFarland optical density was made with 0.85 % NaCl. An appropriate amount of this suspension was added to each well of the API 20NE strip (BioMerieux, Marcy I, Etoile), some of which were covered with mineral oil. The strips were incubated at 30 °C and read at 24 and 48 h. The results were recorded by visual inspection. Positive or negative reactions were recorded for each test cupule, translated into numerical profiles and interpreted with the APILAB PLUS software package as proposed by the manufacturer.

### Antimicrobial susceptibility test

The Kirby–Bauer disk diffusion susceptibility test as described by Wootton *et al*. [23] was used to obtain the antimicrobial resistance profile of the isolates for gentamicin (30 µg), colistin (10 µg), amoxicillin–clavulanic acid (30 µg), cefepime (30 µg), trimethoprim–sulfamethoxazole (25 µg), piperacillin–tazobactam (30 µg), imipenem (10 µg), doxycycline (30 µg) and ceftazidime (30 µg) [23–25]. A strain of *B. thailandensis* confirmed with standard PCR was used as a reference and interpretation of the results was performed according to European Committee on Antimicrobial Susceptibility Testing (EUCAST) breakpoints [26, 27]. Resistance to at least one antimicrobial agent in three or more classes of antimicrobial agents defines multidrug-resistant (MDR) pathogens.

### Statistical analysis

The data were analysed using Microsoft Excel (2016 version) to determine the percentage occurrences/prevalence and the bar charts were made using the ggplot2 package in R (version 4.1.2).

## Results

**Fig. 1.**
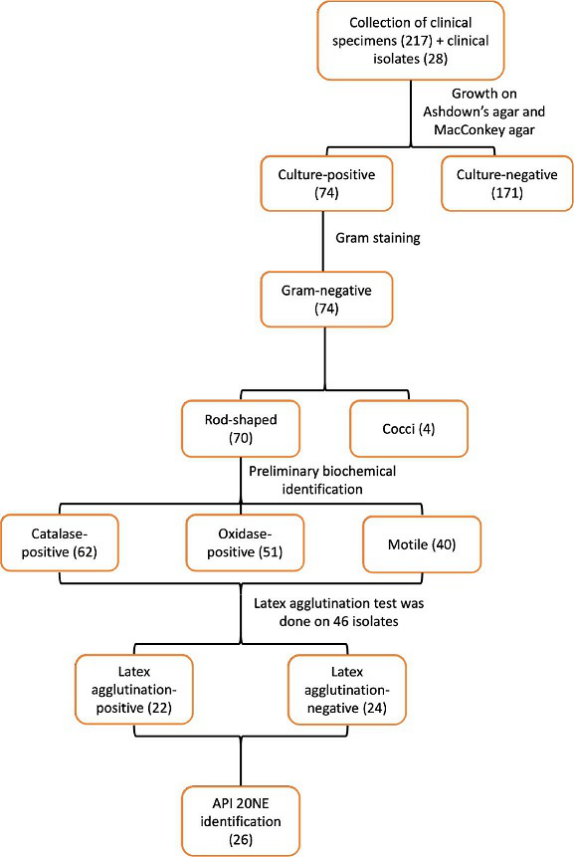
Flow chart showing the phenotypic identification steps and number of samples tested at each stage.

**Fig. 2.**
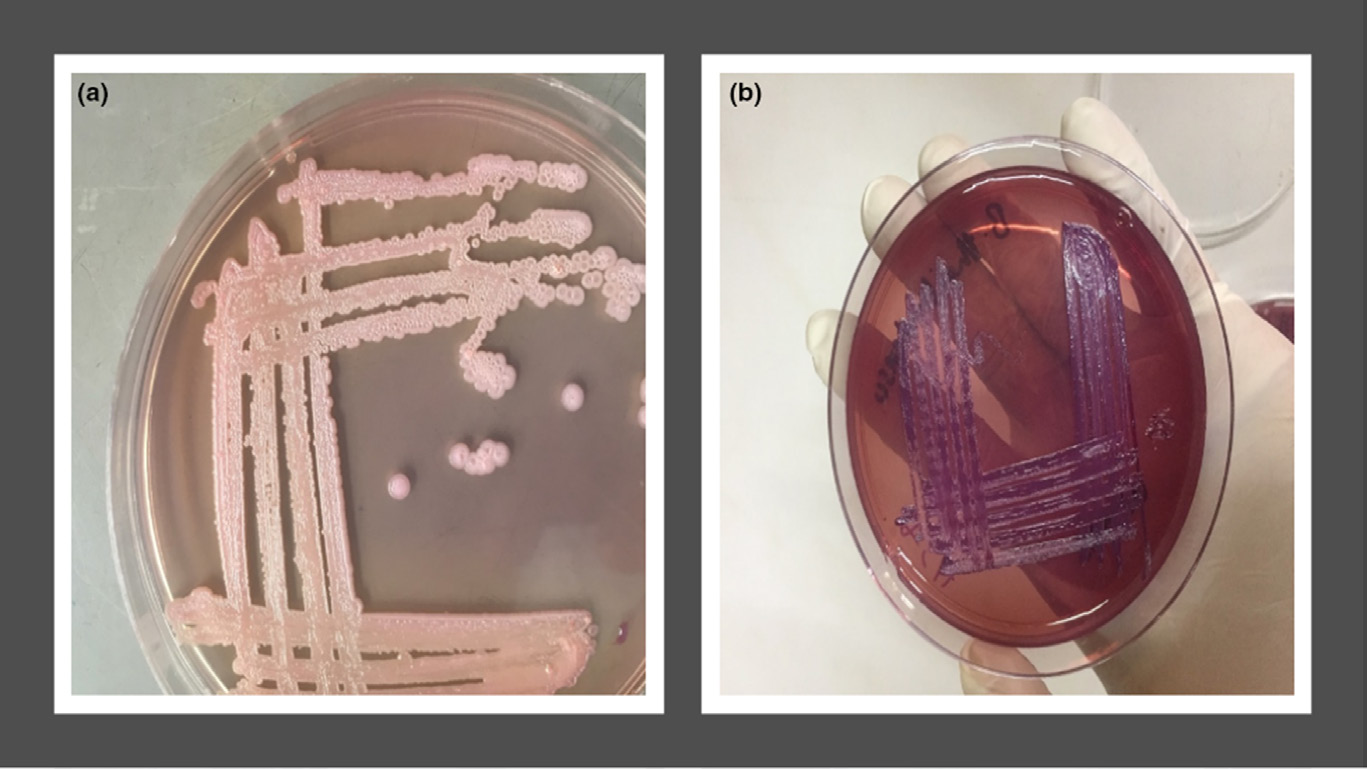
Representative growth on Ashdown’s selective agar plate. (**a**) Shiny appearance of *Burkholderia* isolate. (**b**) *Burkholderia thaillandensis* after 48 h incubation at 37 °C.

**Fig. 3.**
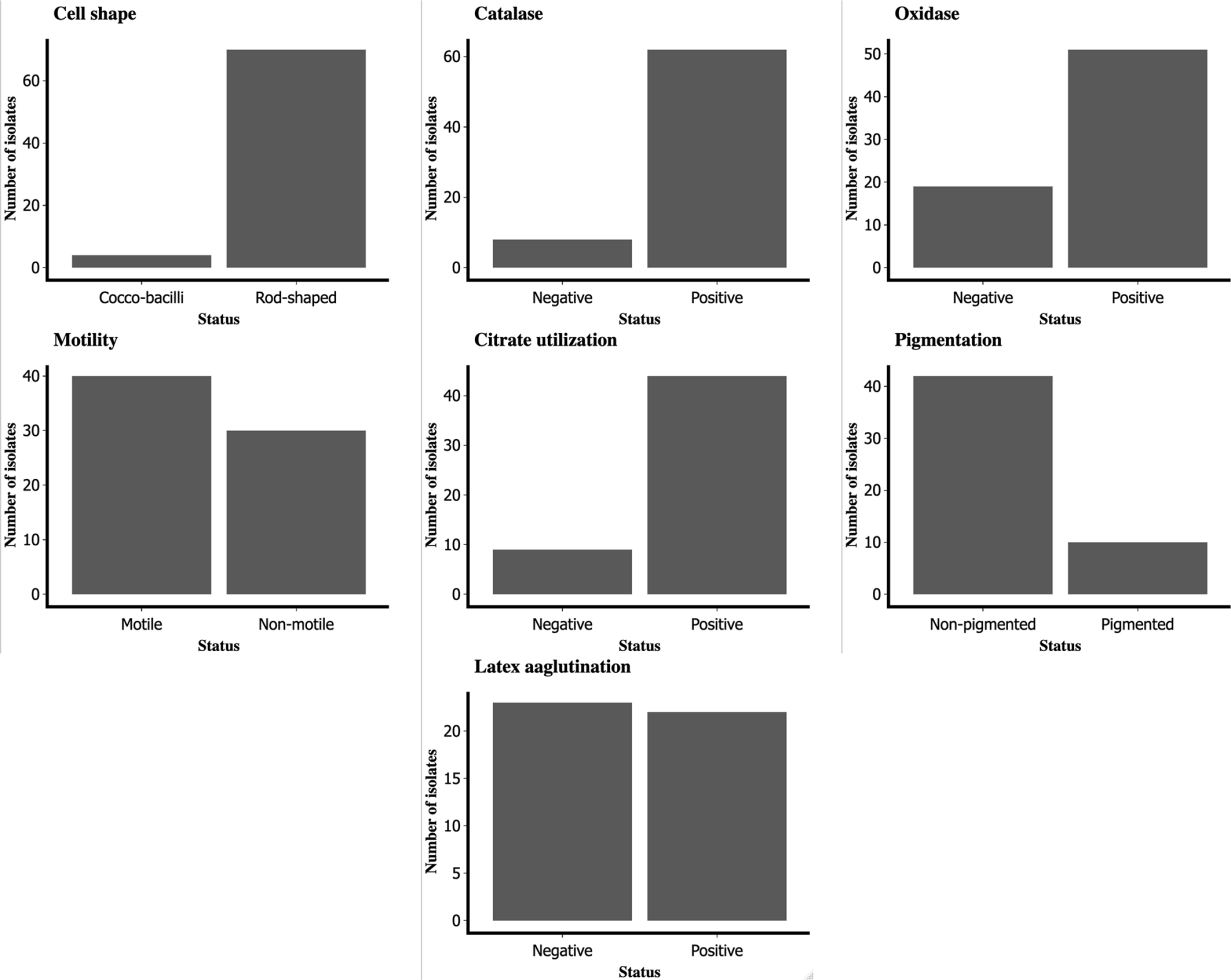
Biochemical assays for characterization of bacterial pathogens from Ashdown’s selective agar. The isolates are grouped into positive and negative.

Cultures of 217 clinical specimens (Data S1) yielded 67 cases of positive growth as non-lactose fermenters on MacConkey agar and typical characteristics on Ashdown’s selective agar (Figs 1 and 2). Seven of the 28 Gram negative non-lactose-fermenting bacilli grew on Ashdown’s selective agar (ASA). In all, 74 isolates were obtained using Ashdown’s selective agar. All culture-positive isolates on Ashdown’s media were confirmed to be Gram-negative through Gram staining and microscopy. Seventy of the isolates (70/74; 94.6 %) were rod-shaped and the remaining isolates had the appearance of coccobacilli when viewed under the microscope. Sixty-two of these organisms (62/70; 88.57 %) were catalase-positive, while 51 of these organisms (51/70; 72.9 %) were oxidase-positive and only 40 organisms (40/70; 57.1 %) showed positivity in motility testing and showed the presumptive features of *Burkholderia *. Forty motile isolates with 6 uncertain isolates were subjected to latex agglutination reaction for *B. pseudomallei* exopolysaccharide; 22 showed agglutination against the monoclonal antibody (Fig. 3).

**Fig. 4.**
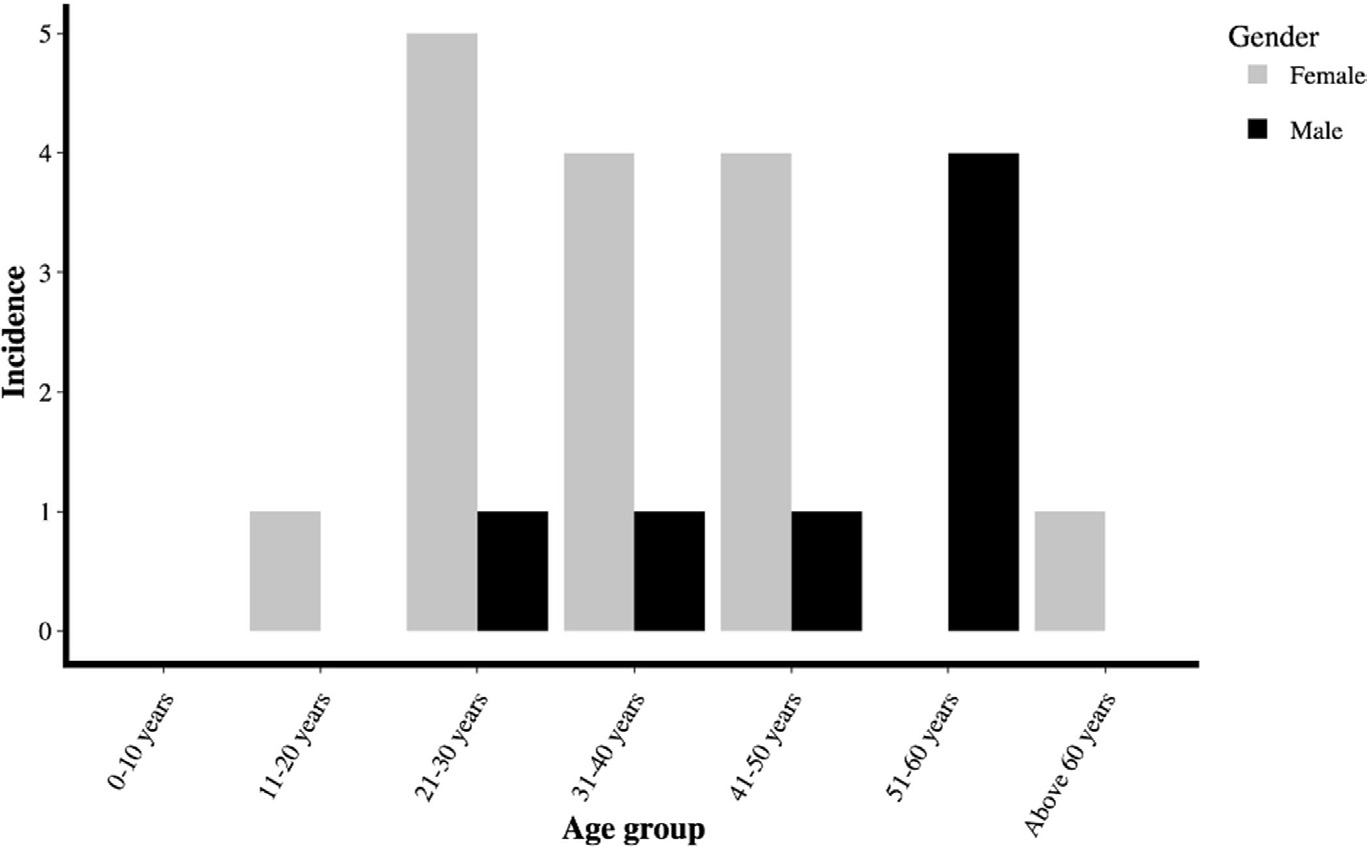
Gender and age distribution of patients for clinical specimens from which isolates originated.

The majority (15; 68.18 %) of the isolates were from female patients. The largest number of isolates (*n*=6) was from people within the age range 21–30, followed by people in the 31–40 (*n*=5) and 41–50 (*n*=5) age groups. Four samples were from patients in the 51–60 age group. Lastly, one isolate each was obtained from the age groups 11–20 and above the age of 60, while none were from the 0–10 age group (Fig. 4).

**Fig. 5.**
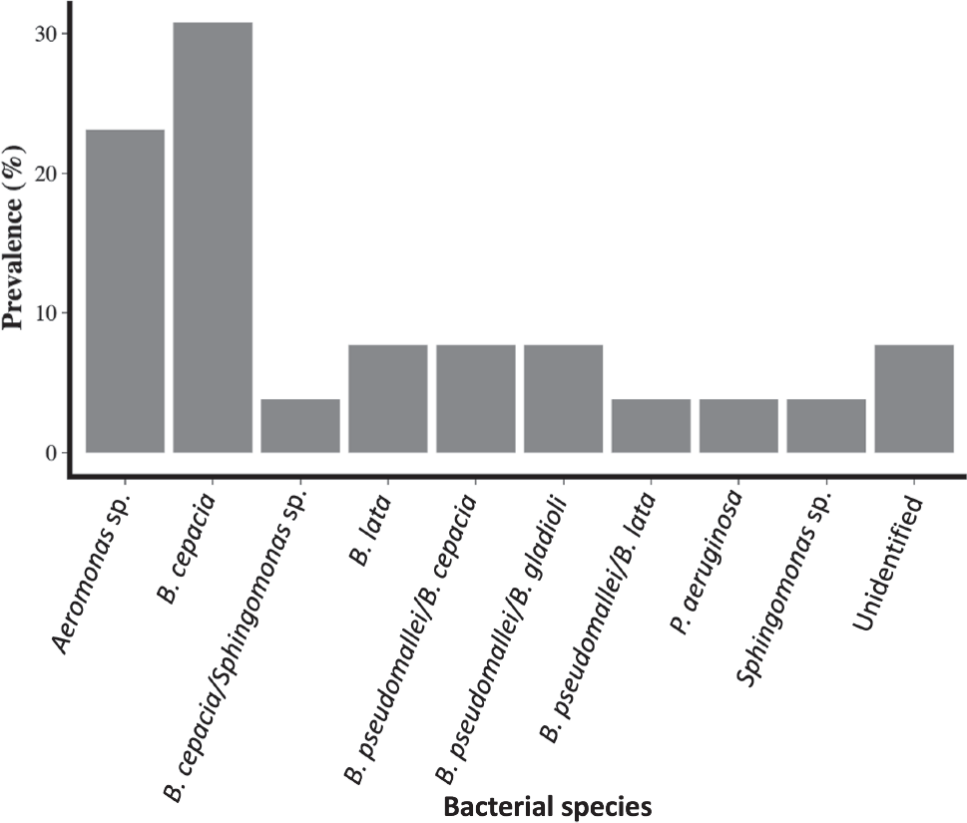
Rate of occurrence of *Burkholderia* and non-* Burkholderia* species identified using analytic profile index (API) 20.

Twenty-six isolates were identified using API 20NE (22 with a certain latex agglutination reaction and additional 4 with an uncertain latex reaction): *B. cepacia* (8/26; 30.8 %), *Aeromonas* s*p*. (6/26; 19.2 %), *Burkholderia lata* (2/26; 7.7 %), *Sphingomonas* sp. (1/26; 3.8 %) and *P. aeruginosa* (1/26; 3.8 %) (Fig. 5). In some case, the kit could not differentiate *B. pseudomallei* from *B. cepacia *, *B. pseudomallei* from *B. lata *, *B. pseudomallei* from *Burkholderia gladioli, B. cepacia* from *B. gladioli* and *B. cepacia* from *Sphingomonas* sp. Overall, the predominant species was *B. cepacia *, at 30.8 % (ID similarity indices: 81.9–98.1 %) and two isolates were unidentifiable (Fig. 3). The majority of the isolates recognized as *Burkholderia* species were from sputum samples (13/161; 8.1 %) followed by throat swab samples (2/24; 8.3 %) and then urine samples (1/60; 1.7 %).

The antibiotic susceptibility profiles for the bacterial isolates to nine different antimicrobials are shown in Table 1. The highest level of resistance was observed towards gentamicin (CN) and colistin (CT) (100 %). The resistance to other antibiotics was as follows; amoxicillin–clavulanic acid (AMC), 71.4 %; cefepime (FEP), 33.3 %; trimethoprim–sulfamethoxazole (W), 38.1 %; piperacillin–tazobactam (TZP), 33.3 %. The multiple resistance phenotypes of the identified *Burkholderia* species revealed that 13 (92.8 %) isolates were resistant to various combination of antibiotics, ranging from four to nine (Table 2). The predominant profile consisting of nine antibiotics (AMC–CN–CT–FEP–W–TZP–IMI–DXT–CAZ) was observed among two strains.

**Table 1.** Antimicrobial susceptibility profiles of *Burkholderia* species isolated from clinical specimens

S/N	ISOLATE CODE	ORGANISM	Antimicrobial agents*								
			AMC	CN	CT	FEP	w	TZP	IMI	DXT	CAZ
**1**.	SR 4087	* Burkholderia cepacia *	0 R	0 R	12 R	12 R	0 R	21 R	0 R	0 R	0 R
**2**.	SR OS	*Burkholderia cepacia/Sphingomonas*	11 R	0 R	9 R	12 R	0 R	35 I	40 S	37 I	25 I
**3.**	SR OLG	Burkholderia lata	0 R	18 R	11 R	28 I	23 I	10 I	0 R	10 R	22 I
**4.**	L155	* Burkholderia */* B. gladioli *	24 I	20 R	14 R	14 R	22 I	35 I	16 R	30 I	0 R
**5**.	L47	*Burkholderia/B. psuedomallei*	0 R	0 R	12 R	27 I	0 R	27 I	32 S	17 R	19 I
**6**.	L114	* Burkholderia cepacia *	0 R	20 R	11 R	27 I	0 R	19 R	27 R	24 I	17 R
**7**.	L115	* Burkholderia cepacia *	0 R	18 R	12 R	18 R	0 R	20 R	14 R	10 R	0 R
**8**.	L124	*Burkholderia/B. pseudomallei*	0 R	0 R	0 R	28 I	0 R	18 R	20 R	12 R	17 R
**9**.	L124c	*Burkholderia/B. gladioli*	22 I	0 R	0 R	28 I	0 R	18 R	17 R	9 R	14 R
**10**.	L118	* Burkholderia lata *	18 R	16 R	10 R	22 I	24 I	27 I	40 S	27 I	15 R
**11**.	BSA	*Burkholderia pseudomallei/B. cepacia*	29 I	26 R	12 R	19 I	25 I	22 I	30 S	34 I	24 I
**12**.	L112	* Burkholderia cepacia *	0 R	0 R	0 R	28 I	0 R	20 R	0 R	10 R	22 I
**13**.	L103	* Burkholderia cepacia *	0 R	19 R	12 R	28 I	23 I	21 R	22 R	15 R	0 R
**14**.	L123	* Burkholderia cepacia *	0 R	19 R	13 R	21 I	0 R	26 I	18 R	0 R	27 I

*Zones of inhibition are in mm.

AMC, amoxicillin–clavulanic acid; CAZ, ceftazidime; CN, gentamicin; CT, colistin; DXT, doxycycline; FEP, cefepime; I, intermediate; IMI, imipenem; R, resistant; S, susceptible; TZP, piperacillin–tazobactam; W, trimethoprim–sulfamethoxazole.

**Table 2.** Multidrug resistance profiles for *Burkholderia* isolates

No. of antimicrobial classes	Resistance pattern	No. of isolates
**2**	CN–CT	1
**4**	AMC–CN–CT–CAZ	1
	CN–CT–FEP–IMI–CAZ	1
		
**5**	AMC–CN–CT–FEP–W	1
	AMC–CN–CT–IMI–DXT	1
	AMC–CN–CT–W–DXT	1
		
**6**	AMC–CN–CT–W–IMI–DXT	1
		
**7**	CN–CT–W–TZP–IMI–DXT–CAZ	1
	AMC–CN–CT–W–TZP–IMI–CAZ	1
	AMC–CN–CT–W–TZP–IMI–DXT–	1
	AMC–CN–CT–TZP–IMI–DXT–CAZ	1
		
**8**	AMC–CN–CT–FEP–W–TZP–IMI–DXT–CAZ	2
	AMC–CN–CT–W–TZP–IMI–DXT–CAZ	1

AMC, amoxicillin–clavulanic acid; CAZ, ceftazidime; CN, gentamicin; CT, colistin; DXT, doxycycline; FEP, cefepime; IMI, imipenem; TZP, piperacillin–tazobactam; W, trimethoprim–sulfamethoxazole.

## Discussion

In this study, the possible existence of *B. pseudomallei* in the samples analysed underscores the need to strengthen laboratory capacity for the identification of this pathogen in the clinical microbiology laboratory. Although each of the phenotypic systems utilized was based on the previous evaluation of expressed properties of *Burkholderia* species, the findings reported here demonstrate that the assays yielded imprecise outcomes for the identification of *B. pseudomallei *. Ashdown’s selective media have been in long-standing use in the literature for the isolation of *B. pseudomallei* [18]. In this study, the overall culture-positive rate for *B. pseudomallei-*like characteristics was 57.1 %. This is inconsistent with the 5.0 % reported by Schully *et al*. [28] We observed that the Ashdown’s selective medium clearly supported the growth of certain Gram-negative organisms with morphological traits that were comparable to those of *B. pseudomallei *. These findings are in line with the experiences of other authors [29]. Hence, for laboratories unaccustomed to working routinely with these organisms, this may be diagnostically challenging.

In this investigation, when compared to a control latex, the antibody–latex suspension recognized 50 %(22/44) of the isolates as positive. In clinical settings, monoclonal antibody-based latex agglutination tests have been shown to be effective in detecting *B. pseudomallei* [30, 31]. This latex agglutination test was evaluated against an inclusiveness panel of 77 *B. pseudomallei* isolates and 76 (98.7 % sensitivity) of these were positive [31]. However, members of the *B. cepacia* complex (Bcc) have been shown to cross-react with *B. pseudomallei *-specific latex agglutination [32]. This observation was confirmed when our isolates were subjected to API 20NE analysis.

Although commercial biochemical panels and nucleic acid amplification techniques have made it possible for diagnostic laboratories to identify most bacterial pathogens with high accuracy and speed, their availability in low-resource settings remains limited, particularly for unusual opportunistic pathogens such as *Burkholderia* species. Nonetheless, the usefulness of biochemical identification techniques is restricted by the requirement for further tests in order to reliably identify strains to the species level, as well as the nonexistence of some species in the relevant databases [29, 33]. Whilst some authors have attested to the misidentification tendencies of the API 20NE system [20], it can be constructively suggested that API 20NE has good mechanisms for reliably recognizing *B. pseudomallei *., [18, 30]

In several studies, the accuracy of API 20NE for the identification of *B. pseudomallei* ranged from 37 to 98 % [30, 33]. In this study, none of the 26 isolates was distinctly identified as *B. pseudomallei* and 4 isolates appeared to have an alternative similarity index as *B. lata *, *B. gladioli* and *B. cepacia *. These non-* B. pseudomallei* species can cause melioidosis-like symptoms and share properties with *B. pseudomallei* at the biochemical level, which could make species-level identification with biochemical panels such as API 20NE challenging. *B. gladioli* as well as its more distantly related Bcc family, *B. cepacia* and *B. lata *, may survive in the same ecological niches as *B. pseudomallei. B. gladioli* are predominately environmental species and have been implicated in a variety of opportunistic infections, including lung infections [21]. Nonetheless, the bulk of our isolates were recognized as *B. cepacia *, a well-known Bcc member. Given the fact that the global burden of Bcc infections is unknown, members of the group have been documented to infect immunocompromised individuals and drug users [22, 34].

In the present paper, *Aeromonas* occurred at a high proportion, with an identity probability of 82.5–91.2 %. Other non-fermentative Gram-negative bacilli (* P. aeruginosa* and *Sphingomonas* sp.) were also found, indicating that these bacteria have high survival potential in hospital environments. Due to their perceived ability to develop resistance to several classes of antibiotics, they have become an issue of public health concern, particularly among critically ill patients [27, 35, 36]. The highest frequency of *Burkholderia* species was found in sputum, with a total percentage of 61.5 % (16/26), in this investigation. This is higher than the findings of Al-Dahash *et al.* [27], who found 1.6 % *B. cepacia* among 250 sputa analysed. It is also inconsistent with the findings of Linju *et al.* [27]. Females were found to have a greater number of *Burkholderia* (62.96 %). This contradicts the results of Linju *et al.* [37], which showed male dominance, but similar to data from Dizbay *et al.* [38] on *B. cepacia *.

This study further buttresses previous reports showing that the use of only phenotypic assays to identify *B. pseudomallei* in clinical settings might not be sufficient [20, 39, 40]. Greer *et al*. [39] reported the misidentification of *B. pseudmallei* as *Acinetobacter* spp. in their studies. Zong *et al*. [40] also reported that the VITEK identification system misidentifies *B. pseudomallei* as *B. cepacia *. Similarly, Wu *et al*. [41] reported the misidentification of *B. pseudomallei* as *Aeromonas sobria *, *Alcaligenes faecalis *, *Pseudomonas fluorescens* and *Burkholderia thaillandensis* using Vitek 2 Compact, Phoenix, Vitek MS and API 20NE, respectively. Since culturing and phenotypic detection systems such as Vitek and API 20NE are the most common identification assays in many clinical laboratories in Nigeria, it might be difficult to diagnose melioidosis accurately in Nigeria. As a result, there is need to invest in molecular diagnostic tools for effective and accurate clinical diagnosis in the country.

The high prevalence of antimicrobial drug resistance observed among our isolates is alarming and indicates the antibiotic-resistant nature of *Burkholderia *, which could make the management of associated infections demanding. The antibiotic susceptibility results revealed that the organisms showed 100 % resistance to gentamicin and colistin. The lowest resistance was to cefepime and piperacillin–tazobactam. Similar findings were reported by Khosravi *et al*. [42], who observed that *B. pseudomallei* from clinical and environmental samples were resistant to multiple antibiotics. On the contrary, Linju *et al*. [37] described good susceptibility of *Burkholderia* sp. to ceftazidime, which has been described as the drug of choice for *B. pseudomallei* infections [29]. While Nhung *et al*. [29] recorded 100 % susceptibility to ceftazidime, imipenem and amoxicillin-clavulanic acid among their *B. pseudomallei* and *B. cepacia* isolates, elevated resistance to other antibiotics has been observed by other authors [37]. This disparity could be due to the variety of strains collected or perhaps the misuse of antibiotics in Nigeria.

The current research has certain limitations. The first is that the assays were selected and utilized based on the information available in the literature. Secondly, we did not re-evaluate the results for reproducibility for our isolates due to short supply of consumables. In addition, we did not have access to a standard *B. pseudomallei* strain that could be used as a positive control due to safety reasons. The organism has been classified as a potential bioterrorism organism and is not easily obtained, especially in Nigeria. However, it is noteworthy that the finding suggests the presence of *B. pseudomallei* in clinical settings and confirms the occurrence of other *Burkholderia* species. Furthermore, molecular techniques such as 16S rRNA sequencing and whole-genome sequencing would have helped to confirm the identity of the organisms, although we could not use any of these techniques due to resource constraints. Although the clinical implications have not been established, and nor is there a protocol to identify the infection in our setting, scaling up of laboratory capacity is very urgently needed since further studies using genotypic assays will have significant implications for public health.

## Conclusion

The limited ability of the phenotypic methods to provide definitive identification of *B. pseudomallei* was notable. This indicates that misidentification of this organism in hospitals and the associated infections could have been occurring due to under-reporting and poor patient management. The multidrug-resistant nature of the isolates in this study, especially to antibiotics that are often used as drugs of choice for the treatment of bacterial infections, including melioidosis, is a great public health concern. Hence, there is a need for more epidemiological studies on the occurrence and antibiotic resistance of *B. pseudomallei* in Nigeria, which would include molecular techniques. Efforts should be made to raise awareness and diagnostic competence, as well as to stimulate case reporting. Clinicians would benefit from having thorough epidemiological data on the organism for the evaluation of melioidosis cases. It is also imperative that clinicians and medical laboratory scientists should be aware of shifting bacterial epidemiology and rising antimicrobial resistance, especially in organisms that are not routinely checked for. This might help health officials and policymakers to implement monitoring, mitigation and preventive efforts for curtailing antimicrobial resistance.

## Supplementary Data

Supplementary material 1
